# A cross-correction gene therapy approach for CDKL5 deficiency disorder improves the pathological phenotype of CDD patient-derived cortical organoids

**DOI:** 10.3389/fbioe.2025.1744903

**Published:** 2026-01-21

**Authors:** Giorgio Medici, Angelica M. Bove, Stefania Trazzi, Francesca Puppo, Manuela Loi, Nicola Mottolese, Giulia Candini, Federica Trebbi, Sandra Sanchez, Alysson R. Muotri, Elisabetta Ciani

**Affiliations:** 1 Department of Biomedical and Neuromotor Sciences, University of Bologna, Bologna, Italy; 2 Departments of Pediatrics and Cellular and Molecular Medicine, University of California San Diego, La Jolla, CA, United States

**Keywords:** CDD, CDKL5, CNS disorders, cortical organoids, cross-correction, gene therapy

## Abstract

Efficient delivery of biological material to the central nervous system remains a key limitation of conventional gene therapies. Recently, we developed a novel strategy based on a secretable and cell-penetrating TATk-CDKL5 fused protein which enhances the brain biodistribution and the therapeutic efficiency of the gene therapy approach in a mouse model of CDKL5 Deficiency Disorder (CDD). Here, to compare the efficacy of the TATk-CDKL5 gene therapy with a conventional approach in correcting the CDKL5 Deficiency Disorder pathological phenotype, we employed cortical organoids generated from CDD patient-derived iPSCs as a human model of CDD. We found greater therapeutic efficacy of the recombinant TATk-CDKL5 protein compared to the CDKL5 protein alone in improving or ameliorating defects caused by the absence of CDKL5, such as abnormal hyperexcitability evaluated with microelectrode arrays (MEA). Interestingly, CDD cortical organoids exhibited reduced cell proliferation and increased neuronal cell death compared to control cortical organoids; defects that were only restored by the expression of the recombinant TATk-CDKL5 protein. Based on the results from phenotypic and functional readouts, these findings suggest that gene therapy using a cross-correction approach offers superior efficiency in treating CDD.

## 1 Introduction

CDKL5 Deficiency Disorder (CDD) is a severe neurodevelopmental condition caused by mutations in the *CDKL5* gene, which encodes a serine-threonine kinase critical for brain development and function (Leonard et al., 2022; Van Bergen et al., 2022). This rare disorder manifests as early-onset epileptic encephalopathy, severe intellectual disability, and impaired motor and visual functions (Leonard et al., 2022; Van Bergen et al., 2022; Kadam et al., 2019; Demarest S. et al., 2019; Jakimiec et al., 2020). Murine models of CDD, including *Cdkl5*-knockout (KO) mice, do not fully recapitulate the early-onset epilepsy but do exhibit several neurobehavioral phenotypes reminiscent of the human disease, including motor coordination deficits, hyperactivity, increased autism-like behaviors, and impaired learning and memory (Amendola et al., 2014; Wang et al., 2012; Okuda et al., 2018; Tang et al., 2019; Yennawar et al., 2019). These models have been instrumental in characterizing the functions of the CDKL5 protein, revealing its pivotal role in neuronal proliferation, differentiation, and function to ensure proper brain development and the establishment of functional neural circuits (Van Bergen et al., 2022; Katayama et al., 2020; Demarest ST. et al., 2019; Kind and Bird, 2021; Fuchs et al., 2014; Fuchs et al., 2019; Loi et al., 2020). Nevertheless, despite significant progress in understanding its molecular basis, no effective therapies are currently available, underscoring the urgent need for innovative approaches to mitigate the debilitating symptoms of CDD (Hong et al., 2022).

In the past decade, treatment approaches centered on complementing affected cells with a functional protein or gene copy have emerged as an encouraging therapeutic strategy for genetic pathologies, especially for monogenic disorders (Wang et al., 2021; Zhang and Wu, 2024). Over the past few years, several studies from our group and others have demonstrated that gene and protein replacement therapies are promising strategies to treat CDD at the pre-clinical level (Medici et al., 2022; Gao et al., 2020; Voronin et al., 2024; Trazzi et al., 2018). However, efficient delivery of biological material to the central nervous system (CNS) remains a significant challenge when developing effective therapeutic strategies for disorders with widespread neuropathology affecting multiple brain regions (Puhl et al., 2019; Begley, 2004; Cogill et al., 2024). We have recently developed a novel gene therapy approach based on a secretable, cell-penetrating CDKL5 protein (Igk-TATk-CDKL5) (Medici et al., 2022). This therapeutic strategy leverages the ability of the Igk-TATk fusion peptide to enable cellular cross-correction, thereby overcoming the challenges of limited biodistribution and neuronal uptake associated with conventional gene therapy. In a recent preclinical study in *Cdkl5*-KO mice, we have demonstrated that this innovative approach significantly improved the bioavailability and therapeutic efficacy of the CDKL5 protein in the brain, leading to an amplified rescue of pathological phenotypes (Medici et al., 2022).

Yet, it is crucial to evaluate the efficacy of this approach in humanized models that more accurately reflect the disease state to further prove the potential of this promising therapy. As a developmental epileptic encephalopathy, the most prominent and presenting feature of CDD is the early onset of seizures. As mentioned before, most *Cdkl5*-KO mice have shown a lack of spontaneous seizures during early development (Fallah and Eubanks, 2020). Therefore, while murine models have been invaluable for modeling CDD, studying disease mechanisms, and testing therapies, they do not fully replicate the human condition, representing a major limitation.

Advances in stem cell technology have revolutionized disease modeling, offering unprecedented opportunities to study human neurodevelopmental disorders *in vitro*. Human-induced pluripotent stem cells (iPSCs) can generate three-dimensional (3D) brain organoids that mimic the structural and functional complexity of the human brain (Swingler et al., 2023; Ei et al., 2022). These organoids replicate key aspects of normal brain development and recapitulate pathological features observed in patients. Organoids derived from CDD patients display multiple functional and structural derangements underlying CDD neuropathophysiology, including morphological changes and alterations in neuronal connectivity and excitability, thus offering a deeper insight into the disease pathophysiology (Negraes et al., 2021; Wu et al., 2022). Remarkably, CDD organoids exhibit neuronal network alterations, including signs of hyperexcitability and increased synchronization, which may be indicative of the epileptic-like activity characteristic of CDD. While these findings require further validation and characterization, they offer a valuable opportunity to explore seizure-related network dysfunction in a human-derived system, potentially overcoming the limitations of murine models that do not replicate early-onset seizures. By recapitulating multiple cellular and network-level alterations observed in patients, CDD organoids offer a 3D *in vitro* system that bridges the gap between preclinical animal studies and clinical applications, providing an ideal platform to further evaluate the therapeutic potential of novel treatments in a patient-specific context. Therefore, in this study, we investigated the therapeutic potential of the TATk-CDKL5-based gene therapy in CDD patient-derived cortical organoids. Specifically, we compared the efficacy of the secretable TATk-CDKL5 fusion protein with that of the conventional CDKL5 protein. By leveraging the pathological relevance and human specificity of the organoid model, we aimed to provide compelling evidence for the clinical applicability of a gene therapy approach based on a cross-correction mechanism.

## 2 Materials and methods

### 2.1 Generation of cortical organoids and culture

All results presented in this study are based on two independent CDD patient-derived iPSC lines, each with its corresponding isogenic control. Each iPSC line was differentiated into cortical organoids in two independent differentiation batches, which were treated as independent biological replicates. CDD cortical organoids were generated from two patient-derived iPSC lines harboring the same CDKL5 loss-of-function mutation (R59X nonsense mutation) previously described in (Negraes et al., 2021). These iPSC lines are considered functionally full knockouts, as confirmed by the complete absence of CDKL5 protein expression. The organoids were generated following the same protocol as in previous studies (Negraes et al., 2021; Fitzgerald et al., 2024; Trujillo et al., 2019), to ensure consistency in the experimental approach. Specifically, the patient-derived iPSC lines harboring the CDKL5 R59X nonsense mutation correspond to the CDD1 and CDD4 lines in [30], while the control iPSC lines, derived from first-degree sex-matched healthy relatives, correspond to Control1 and Control4 (Negraes et al., 2021). Briefly, iPSC colonies were dissociated using Accutase (Thermo Fisher Scientific; diluted 1:1 with 1X PBS) and plated into low-attachment 6-well plates at an optimized seeding density (∼4 million cells per well) to promote the formation of uniform embryoid bodies (EBs) under continuous agitation on an orbital shaker. Neural induction was initiated by switching to M1 medium consisting of Neurobasal medium (Thermo Fisher Scientific) supplemented with GlutaMAX (Thermo Fisher Scientific), 1% Gem21 NeuroPlex (Gemini Bio-Products), 1% N2 NeuroPlex (Gemini Bio-Products), 1% NEAA (Thermo Fisher Scientific), 1% penicillin/streptomycin (Thermo Fisher Scientific), 10 μM SB431542, and 1 µM dorsomorphin. Key growth factors, including FGF, EGF, and neurotrophic factors (BDNF, GDNF, NT-3), were then added to promote cellular maturation and neural specification. After neural differentiation, cortical organoids were maintained in M2 medium (Neurobasal medium containing GlutaMAX, 1% Gem21, 1% NEAA), with media changes every 3–4 days.

### 2.2 AAV infection

AAVPHP.B expressing the TATk-CDKL5 or the CDKL5 protein were produced by Innovavector srl (Pozzuoli (NA), Italy) using the pAAV_CBh-TATk-CDKL5-HA or pAAV_CBh-CDKL5-HA plasmid, as previously described (Medici et al., 2022).

For AAVPHP.B transduction efficiency analysis, 6-week-old control cortical organoids were plated onto a poly-L-ornithine/laminin-coated glass bottom dish or grown in suspension in a 6-well plate under continuous agitation. At 10 weeks old, cortical organoids were treated with AAVPHP.B virus carrying the Igk-TATk-CDKL5 vector at different doses (7.2*10^9^, 3.6*10^10^, 7.2*10^10^ viral genome (vg)/organoid (org)) or with PBS as control. The medium was changed 48 h post-infection and replaced with 1:1 mixture of fresh M2 media and previously collected conditioned media. Organoids were collected 1 week after infection for immunostaining analysis.

For electrophysiology recordings, 6-week-old CDD and control cortical organoids were plated onto polyethyleneimine (PEI)/laminin-coated 48-well microelectrode array (MEA) plates (three organoids per well). At 11 weeks old, cortical organoids were treated with AAVPHP.B virus carrying either the CDKL5 or the Igk-TATk-CDKL5 vector at the dose of 3.6*10^10^ vg/org, or with PBS as a control. Three weeks post infection, organoids were collected for Western blot analysis.

For immunofluorescence staining, 6-week-old CDD and control cortical organoids were grown in suspension in a 6-well plate under constant agitation. At 11 weeks old, cortical organoids were treated with AAVPHP.B virus carrying either the CDKL5 or the Igk-TATk-CDKL5 vector at the dose of 3.6*10^10^ vg/org, or with PBS. Organoids were collected 3 weeks post-infection for immunohistochemistry.

### 2.3 Electrophysiological recordings

MEA recordings were conducted in complete BrainPhys medium (BrainPhys supplemented with SM1, StemCell Technologies), which was introduced 1 day before recordings. Following data acquisition, the medium was replaced with M2 medium for long-term maintenance on MEA plates, with half-medium changes performed twice per week. MEA electrophysiological recordings were performed once per week using the Maestro MEA system and the AxIS Software Spontaneous Neural Configuration (Axion Biosystems) with a band-pass filter of 200 Hz and 3 kHz cutoff frequencies. Briefly, spikes were detected with AxIS software using an adaptive threshold crossing set to 5.5 times the standard deviation of the estimated noise for each electrode (channel). Electrodes that detected at least five spikes/min were classified as active electrodes using Axion BioSystems’ Neural Metrics Tool. Bursts were identified in the data recorded from each individual electrode using an inter-spike interval (ISI) threshold that requires a minimum of five spikes with a maximum ISI of 100 ms. Network bursts in the well required at least ten spikes under the same ISI with a minimum of 25% active electrodes. The synchrony index was calculated using a cross-correlogram synchrony window of 20 ms.

Organoids derived from different CDD iPSC lines were never pooled within the same MEA well. Each well was line specific, containing three organoids originating from a single iPSC line of a single differentiation batch. Because no line-dependent effects were detected, data from the two CDD lines were pooled at the analysis stage to increase statistical power and provide a more generalizable assessment of treatment effects.

MEA recordings were performed on intact organoids grown directly on MEA plates, thus primarily capturing electrophysiological activity from the outer, superficial layers of the organoids, which are more accessible and better transduced.

### 2.4 Western blotting

Organoids grown on the MEA plate for electrophysiological recordings were briefly washed with PBS and detached from the plate using Accutase (Stemcell Technologies). Collected organoids were lysed in ice-cold RIPA buffer (50 mM Tris–HCl pH 7.4, 150 mM NaCl, 1% Triton-X100, 0.5% sodium deoxycholate, 0.1% SDS) supplemented with cOmplete ULTRA mini protease inhibitor (Roche) and PhosSTOP phosphatase inhibitor (Roche). Protein concentration was determined using BCA assay (Walker, 1994). Equivalent amounts of protein (20 μg) were subjected to electrophoresis on a 4%–12% Invitrogen™ Bolt™ Bis-Tris Gel (Invitrogen) and transferred onto nitrocellulose membrane using the iBlot2 dry blotting System (Thermo Fisher). After blocking with 1× TBS + 0.1% Tween-20 (TBS-T) containing 5% BSA for 1 h at room temperature, the membrane was probed with the primary antibody overnight at 4 °C. Next, the membrane was washed 3 times (10 min each) with TBS-T and incubated with the secondary antibody for 2 h at room temperature, followed by 3 more washes in TBS-T. Odyssey CLx imaging system (LICORbio) was used for signal detection, and semi-quantitative analysis was performed using Odyssey Image Studio software (LICORbio). The primary and secondary antibodies used are listed in Supplementary Table S1.

### 2.5 Tissue preparation and immunostaining

Infected organoids grown on glass bottom dishes were washed with 1x PBS, fixed with a 4% paraformaldehyde (PFA) in 100 mM phosphate buffer (pH 7.4) solution for 20 min at RT, and subsequently washed in 1x PBS, before being processed for immunostaining as whole-mount organoids.

Infected organoids grown in suspension were washed with 1x PBS, fixed in a 4% PFA solution for 24 h at 4 °C, and subsequently submerged in a 30% sucrose solution for another 24 h at 4 °C, before being flash frozen in plastic molds containing Tissue-Tek® OCT and stored at −80 °C. Frozen organoids were cut with a cryostat into 15-µm-thick sections that were mounted onto SuperFrost® Plus microscope slides, dried on the bench and stored at −20 °C until being processed for immunostaining.

For immunofluorescence analyses, to avoid heterogeneity introduced by rosette structures predominantly found in outer layers, only sections from the inner core of the organoids were selected for staining and quantification. Outer superficial sections containing rosettes were intentionally discarded to allow more homogeneous fluorescence quantification across the entire analyzed section.

### 2.6 Immunostaining

Organoid slices were thawed and washed twice with 1x PBS to remove OCT. A hydrophobic barrier was drawn around the area with organoid slices. Organoid slices on glass-slide or whole-mount organoids were permeabilized in 1x PBS + 0.1% Triton X-100 (PBS-T) for 10 min and then blocked with PBS-T containing 3% BSA. Before permeabilization and blocking, for slides undergoing PCNA and Cleaved Caspase 3 staining, an additional step of Antigen Retrieval was performed by boiling samples for 10 min in preheated 10 mM Citrate Buffer, followed by extensive washes in 1x PBS. All samples were incubated overnight at 4 °C with primary antibodies diluted in PBS-T + 1% BSA. The following day, slides were washed 3 times in PBS-T and incubated for 2 h with secondary antibodies. After 3 additional washes in PBS-T, all samples were incubated with 4′,6-diamidino-2-phenylindole dihydrochloride (DAPI) for nuclei counterstaining. All samples were mounted using DAPI-fluoromount-g or ProLong Gold Antifade (Thermo Fisher Scientific) and air-dried on bench in the dark. The primary and secondary antibodies used can be found in Supplementary Table S1.

### 2.7 Image acquisition and measurements

Immunofluorescence images were taken with a Nikon Eclipse TE 2000-S inverted microscope equipped with a DS-Qi2 digital SLR camera (Nikon) or with a Zeiss fluorescence microscope equipped with Apotome (Axio Observer Apotome, Zeiss). All images were acquired using the same exposure and gain parameters. The organoid area was manually traced using NIS-Elements AR software (Nikon).

#### 2.7.1 Intensity-based analysis

The fluorescence signal intensity of Ki67, NeuN, or Map2 staining in cortical organoids was quantified starting from ×20 magnification images of organoid slices. For HA fluorescence signal intensity, quantification was performed on images of both whole-mount organoids and organoid sections. All images were acquired using the same exposure and gain parameters. The organoid area was manually traced using NIS-Elements AR software (Nikon). Signal intensity was quantified by determining the fluorescence intensity of all positive (bright) pixels within the traced area. The signal intensity of Ki67, NeuN or Map2 was normalized to DAPI signal intensity of the same organoid. Approximately 6-9 organoids were analyzed for each experimental group.

#### 2.7.2 Cell density

The number of PCNA-, Cleaved Caspase 3- or NeuN-positive cells, and pyknotic nuclei were manually counted using the point tool of the Image Pro Plus software (Media Cybernetics) starting from ×40 magnification images. Proliferation and apoptosis analysis were performed evaluating the percentage of PCNA- or Cleaved Caspase 3-positive cells over the total cell number. The overall cell density of cortical organoids was evaluated as number of DAPI-positive nuclei/area and expressed as number of cells/µm^2^. Apoptotic cells in control and CDD cortical organoids were identified by counting the number of pyknotic nuclei, expressed as percentage of DAPI-positive nuclei. Apoptotic neuronal cells were identified by counting the number of NeuN-positive cells displaying pyknotic, i.e., condensed and fragmented nuclei (Ziegler and Groscurth, 2004) and expressed as percentage of the total NeuN-positive cells. Approximately 3,000–5,000 cells were analyzed from each sample.

#### 2.7.3 Statistical analysis

Statistical analysis was performed using Prism 8.0 software (GraphPad). All data are reported as means ± SEM with dots in each graph representing individual values. The *n* is defined as the unit of data samples with each sample corresponding to a pool of organoids derived from independent patient-derived iPSC lines across two differentiation batches. Statistical values including the *n*, number of organoids, number of cell line, number of independent differentiation batch, statistical test, and significance are reported in the Figure legends. Outlier values were excluded according to the ROUT method (Q = 1%). Gaussian distribution of data sets was tested using D'Agostino-Pearson normality test or Shapiro-Wilk for low sample size. Equality of variances was tested using Brown-Forsythe test. Differences within datasets with normal distribution were analyzed for significance using Fisher’s LSD after ordinary one-way ANOVA when the population had equal variances, or Unpaired t-test after Welch’s ANOVA when unequal variance was present. Datasets with non-parametric distribution were analyzed using uncorrected Dunn’s test after Kruskal–Wallis test. All relevant pairwise statistical comparisons, including CDD vs. TATk-CDKL5 and control vs. TATk-CDKL5, were performed for each dataset; only statistically significant comparisons are shown to avoid figure overcrowding. A probability level of P < 0.05 was considered to be statistically significant. A descriptive statistic of the treatment factor is given in Supplementary Table S2.

## 3 Results

To assess the therapeutic efficacy of a gene therapy approach in a human-based *in vitro* CDD model, iPSCs generated from CDD patients (harboring a nonsense mutation introducing a stop codon at position 59 of the catalytic domain of CDKL5: c.175C>T; p.R59X) were used to generate electrophysiologically-active CDD cortical organoids containing differentiated neurons, as previously described (Negraes et al., 2021; Fitzgerald et al., 2024; Trujillo et al., 2019). Sex-matched iPSCs derived from first-degree relatives of the patients were used to generate healthy control organoids.

**FIGURE 1:**
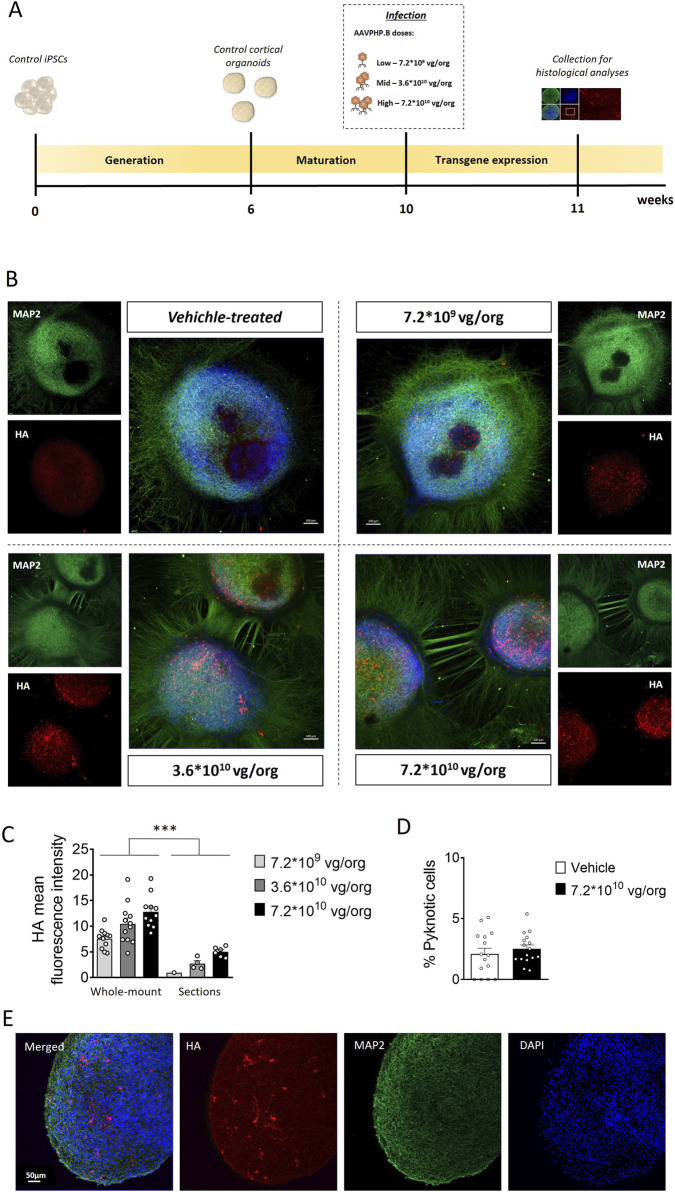
Experimental design and AAVPHP.B transduction efficacy in cortical organoids. **(A)** Schematic timeline showing the generation of control cortical organoids, infection, and subsequent histological analysis for the evaluation of transgene expression. **(B)** Representative fluorescence images of whole-mount immunostaining for CDKL5 (HA, in red) and MAP2 (in green) of 11-week-old control cortical organoids grown on a glass bottom dish, treated with TATk-CDKL5 gene therapy vector at different doses (7.2*10^9^, 3.6*10^10^, 7.2*10^10^ vg/org), or vehicle as a control, and collected 1-week after infection for immunostaining. Scale bar = 100 μm. **(C)** Quantification of HA mean intensity in whole-mount organoids (superficial zone) and organoid slices (inner zone); organoids were infected with different virus doses as described in **(A,B)**. **(D)** Percentage of apoptotic cells in vehicle-treated and high dose viral (7.2*10^10^ vg/org)-treated cortical organoids. Values in **(C)** and **(D)** are presented as mean ± SEM. ***p < 0.001 (Fisher’s LSD after two-way ANOVA for dataset in **(C)**; Unpaired t-test for dataset in **(D)**. **(E)** Representative fluorescence images of a cortical organoid slice showing CDKL5 expression (HA, in red) in control cortical organoids grown under constant agitation, infected with TATk-CDKL5 gene therapy vector at the dose of 7.2*10^10^ vg/org and collected 1-week after infection; MAP2 (in green) and nuclei counterstained with DAPI (in blue). Scale bar = 50 μm.

To optimize transgene expression while maintaining the viability of cortical organoids, we first evaluated the appropriate dose of AAVPHP.B virus. Cortical organoids cultured on glass bottom dishes were infected with three different viral doses (7.2*10^9^, 3.6*10^10^, 7.2*10^10^ viral genome (vg)/organoid (org); Figure 1A), guided by prior experiments using the same AAV serotype (Cho et al., 2022). One-week post-infection, immunofluorescent staining of whole-mount organoids revealed robust CDKL5 expression predominantly localized to the outer layer of the organoids, with the number of CDKL5-positive cells increasing proportionally to the viral dose (Figures 1B,C). CDKL5-positive cells were detected both within rosette-like structures (Figure 1B), which are enriched in neural progenitor cells, and throughout the overall organoid structure, where neurons are the predominant cell type, as shown by MAP2 staining (Figure 1B), indicating transduction of multiple neural cell types, including progenitors and differentiated neurons. To further define the identity of transduced cells, we performed HA/NeuN double immunolabeling 3 weeks post-infection (Supplementary Figure S1B), which showed that approximately 40%–50% of HA-positive cells correspond to mature neurons. Consistent with previous reports on AAV tropism, including AAVPHP.B, in human cortical organoids (Cho et al., 2022; Stanton et al., 2023; Drouyer et al., 2024), HA/CDKL5 immunostaining revealed expression in MAP2^+^ and NeuN^+^ cells (Supplementary Figures S1A,B), as well as in NeuN^−^ cells (Supplementary Figure S1B, left panels, white arrows) and progenitor-enriched rosette regions (Supplementary Figure S1B, right panels). Importantly, organoids subjected to infection, at all viral doses, retained their characteristic rounded morphology, with no signs of necrosis or disaggregation (Figures 1B,E). Quantification of DAPI-positive nuclei with morphological features consistent with cell death revealed no differences between untreated and AAVPHP.B virus-treated organoids (Figure 1D), indicating that neither viral infection nor TATk-CDKL5 expression compromised organoid viability. As expected due to the lack of vascularization, imaging of immunostained organoid sections showed a heterogeneous pattern of transgene expression, with stronger CDKL5 protein detection at the organoid periphery and a lower signal in the core region, consistent with limited viral diffusion, and resulting in partial recovery of CDKL5 expression in only a subset of cells (Figures 1B,E; Supplementary Figure S1A). This spatial restriction explains why whole-mount immunostaining preferentially reflects transgene expression in superficial layers and results in higher HA fluorescence intensity compared with organoid sections (Figure 1C). In this three-dimensional system, intensity-based measurements therefore provide a more biologically meaningful assessment of dose-dependent transgene expression than global estimates of transduction efficiency, which would largely reflect diffusion constraints rather than true viral performance.

Given that high viral doses could lead to excessive infection and potentially mask the efficacy of the cross-correction mechanism, through which recombinant TATk-CDKL5 protein is distributed to non-infected cells, we selected the intermediate viral dose (3.6*10^10^ vg/org) for subsequent experiments to balance expression efficiency and cross-correction potential.

### 3.1 Effect of gene therapy with Igk-TATk-CDKL5 or CDKL5 vector on CDKL5 protein replacement in CDD patient-derived organoids

**FIGURE 2:**
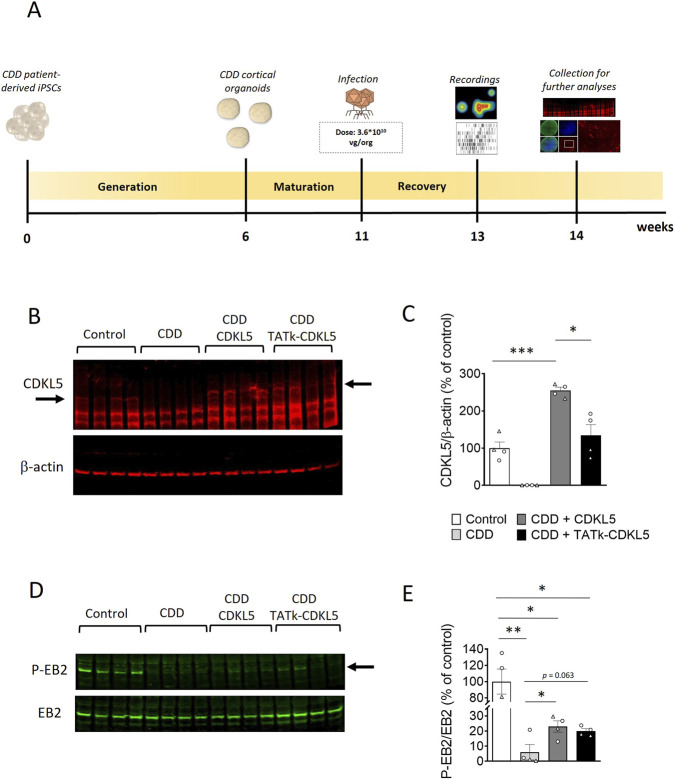
CDKL5 protein replacement in CDD cortical organoids following gene therapy. **(A)** Schematic timeline showing the generation of control and CDD cortical organoids, infection, and subsequent analysis for the evaluation of gene therapy treatments. **(B,C)** Western blot analysis of CDKL5 expression levels in protein extracts from CDD and control cortical organoids grown on MEA plates, treated at 11 weeks old with TATk-CDKL5 or CDKL5 gene therapy vectors at a dose of 3.6*10^10^ vg/org, or vehicle as a control, and collected 3 weeks after treatment for biochemical analysis (Control and CDD conditions: *n* = 4 lanes, 9 pooled organoids per lane, 2 cell lines, 1 differentiation batch). **(B)** Immunoblots of CDKL5 (upper panel) and β-actin (lower panel) proteins. The histogram in **(C)** shows quantification of CDKL5 expression levels normalized to β-actin levels. **(D,E)** Western blot analysis of phosphorylation levels of microtubule end-binding protein EB2 in protein extracts from cortical organoids (Control and CDD conditions: *n* = 4 lanes, 9 pooled organoids per lane, 2 cell lines, 1 differentiation batch). **(D)** Immunoblots of the phosphorylated form of EB2 protein (upper panel) and total protein levels (lower panel). The histogram in **(E)** shows quantification of phosphorylated EB2 protein levels normalized to corresponding total EB2 protein levels. Data sets in **(C)** and **(E)** are expressed as a percentage of vehicle-treated control organoids. Dots and triangles in the scatter plots identify the two independent CDD iPSC lines. Values are represented as means ± SEM. *p < 0.05, **p < 0.01, ***p < 0.001 (Unpaired t-test after Welch’s ANOVA).

Previous findings indicate that increased spike frequency and network hypersynchrony in CDD organoids are confined to early development (12–16 weeks), after which activity gradually converges toward control-like levels (Negraes et al., 2021). Therefore, to compare the efficacy of a cross-correction gene therapy approach with that of a classical approach, a group of CDD organoids (11 weeks old) were infected with an AAVPHP.B viral vector delivering the genetic material for the expression of either the recombinant Igk-TATk-CDKL5 (CDD + TATk-CDKL5) or the native wild-type protein (CDD + CDKL5) at a dose of 3.6*10^10^ vg per organoid (Figure 2A). As a control, a group of CDD and healthy organoids were treated with vehicle.

We performed Western blot and immunohistochemical analyses to evaluate the extent of CDKL5 protein recovery in CDD organoids following viral treatment. As expected, CDKL5 was not detectable in CDD patient-derived cortical organoids (Figures 2B,C). Following viral treatment, CDKL5 protein was expressed at the same level as in controls in TATk-CDKL5-treated organoids or even at higher level in CDKL5-treated organoids (Figures 2B,C), suggesting a complete recovery of CDKL5 expression in CDD organoids. However, immunohistochemical analysis of organoid sections revealed that CDKL5 expression was restricted to a subset of cells, with variable signal intensity, reflecting limited cellular transduction following viral infection (Supplementary Figure S1D). To determine whether the two vectors differed in infection or expression efficiency at the cellular level, we quantified HA fluorescence intensity 3 weeks post-infection. This analysis showed comparable HA signal levels in CDKL5-and TATk-CDKL5–treated organoids, indicating similar transduction and transgene expression efficiency for the two constructs (Supplementary Figure S1C).

To assess the molecular effects of CDKL5 protein re-expression, we examined the functional activation of cellular pathways in which CDKL5 is known to play a direct role. The microtubule end-binding protein EB2 is the most well-known direct CDKL5 phosphorylation target (Silvestre et al., 2024). Accordingly, we found a significant reduction in EB2 phosphorylation levels in vehicle-treated CDD organoids compared to control organoids (Figures 2D,E). Organoids treated with viral vectors showed a marginal increase in EB2 phosphorylation levels in both TATk-CDKL5 and CDKL5 gene therapy-treated organoids (Figures 2D,E). While this increase was slightly significant when evaluated by the pairwise Welch’s t-tests (Figure 2E), a simple unpaired t-test comparison revealed a more significant enhancement compared to untreated CDD organoids (CDD vs. CDKL5 p = 0.034; CDD vs. TATk-CDKL5 p = 0.037). This suggests that the levels of CDKL5 re-expression achieved through viral delivery promote partial re-activation of the EB2 pathway, but are insufficient to restore the pathway to control levels.

### 3.2 Effect of gene therapy with Igk-TATk-CDKL5 or CDKL5 vector on the electrical activity of CDD patient-derived organoids

**FIGURE 3:**
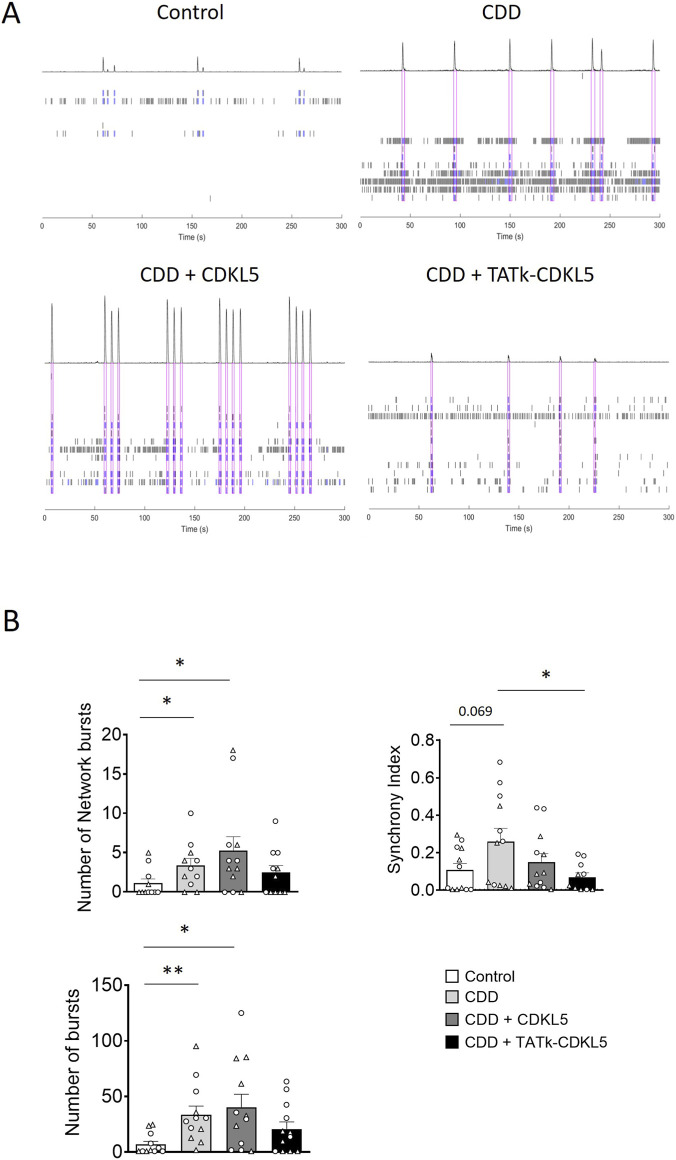
Electrical activity in CDD cortical organoids following gene therapy. **(A)** Representative raster plots of MEA recordings for each condition. **(B)** Electrophysiological recording of CDD and control cortical organoids grown on MEA plates, treated at 11 weeks old with TATk-CDKL5 or CDKL5 gene therapy vectors at a dose of 3.6*10^10^ vg/org, or vehicle as a control. Electrical activity was recorded 2 weeks after treatment. Graphs show the result of several electrical parameters as indicated during 3 min of recording (Control and CDD conditions: *n* = 10–12 wells, 3 organoids per well, 2 cell lines, 1 differentiation batch). Dots and triangles in the scatter plots identify the two independent CDD iPSC lines. Values are represented as means ± SEM. *p < 0.05, **p < 0.01, ***p < 0.001 (Unpaired t-test after Welch’s ANOVA, and Dunn’s test after Kruskal–Wallis for data set “Number of network bursts”).

Cortical organoids exhibit spontaneous electrical activity that reflects the formation and maturation of functional neuronal networks (Trujillo et al., 2019; Tasnim and Liu, 2022; Sharf et al., 2022). As previously shown, analysis of the electrical activity of neural networks demonstrated that cortical organoids lacking CDKL5 functionality are more excitable than control organoids (Negraes et al., 2021), suggesting that CDD neurons are characterized by hyperexcitability, which could underlie the seizures that characterize children with CDD. To evaluate the effect of treatment on the electrical activity of CDD and control organoids, 6-week-old organoids were plated onto a microelectrode array (MEA) plate and monitored weekly to track network activity development. Because planar MEA recordings primarily capture electrophysiological activity from the outer, superficial layers of intact organoids (Huang et al., 2022), the analyses mainly reflect functional changes occurring in these regions. At 11 weeks, organoids were infected, and electrophysiological activity was recorded 2 weeks post-infection (Figures 2A, 3A).

Despite considerable variability in electrical activity across organoids, reflecting their intrinsic heterogeneity, several electrical parameters related to single neurons or neuronal network activity, such as mean firing rate, number of bursts, spikes, and network bursts, significantly differed between control and CDD organoids (Figure 3; Supplementary Figure S2), reaching values even higher than those previously reported in this model (Negraes et al., 2021).

Interestingly, while treatment with the vector delivering native CDKL5 protein was not sufficient to improve or even ameliorate most of the recorded electrical features, treatment with the vector expressing the recombinant TATk-CDKL5 protein showed an increased, although modest, therapeutic effect (Figure 3B; Supplementary Figure S2). Even though the number of spikes and mean firing rate were not significantly rescued (Supplementary Figure S2), a significant amelioration in the number of bursts and network bursts, and a more prominent rescue of the synchrony index was observed (Figure 3B), whereas any apparent worsening of some parameters in CDKL5-treated organoids did not reach statistical significance and reflects increased data dispersion rather than a reproducible detrimental effect.

### 3.3 Effect of gene therapy with Igk-TATk-CDKL5 or CDKL5 vector on cell proliferation of CDD patient-derived organoids

**FIGURE 4:**
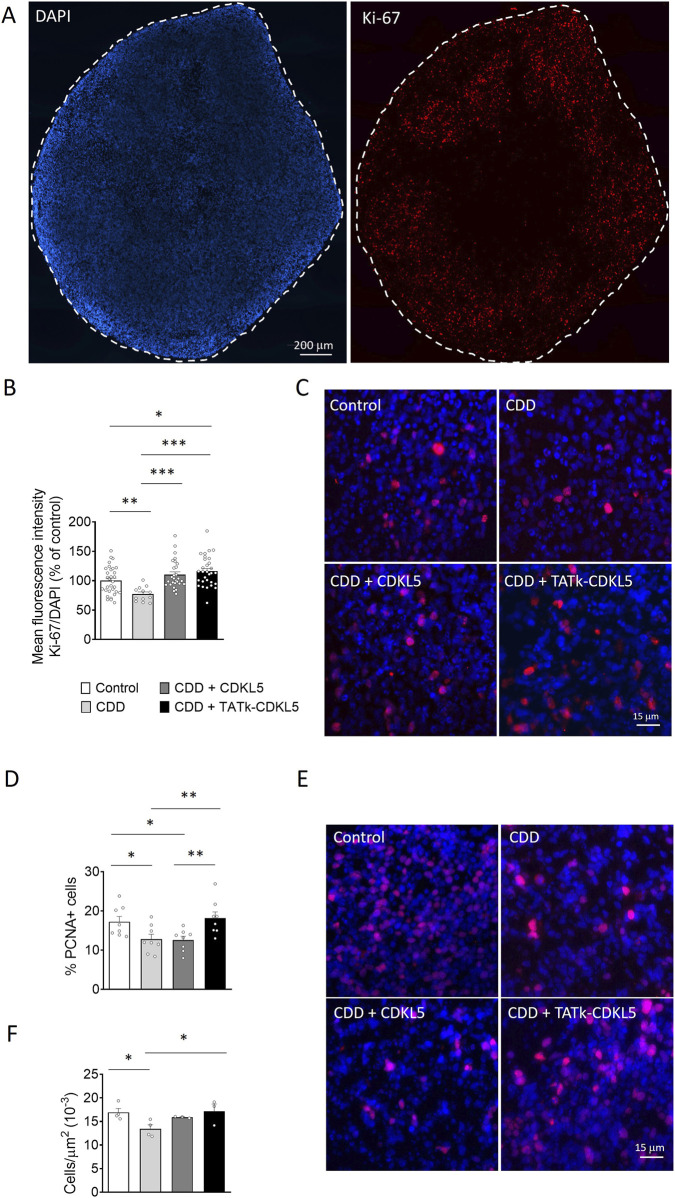
Cell proliferation in CDD cortical organoids following gene therapy. Evaluation of cell proliferation in CDD and control cortical organoids grown under constant agitation, treated at 11 weeks old with TATk-CDKL5 or CDKL5 gene therapy vectors at a dose of 3.6*10^10^ vg/org, or vehicle as a control, and collected 3 weeks after treatment for immunostaining. **(A–C)** Ki-67 immunostaining (Control and CDD conditions: *n* = 15–30 images, from 6-9 organoids, 1 cell line, 2 differentiation batches). Representative fluorescence images **(A)** and high-magnification images **(C)** of a cortical organoid slice immunolabeled for proliferation marker Ki-67 (in red) and counterstained for cell nuclei with DAPI (in blue). Dotted lines indicate the manually traced area of the organoid in which signal intensity was quantified. Scale bar = 200 μm (low-magnification); 15 μm (high-magnification). Quantification of Ki-67 intensity normalized to DAPI intensity per organoid area **(B)**. Data are expressed as a percentage of vehicle-treated control organoids. **(D,E)** Quantification of PCNA-positive cells in cortical organoids treated with gene therapy vectors or vehicle as a control (Control and CDD conditions: *n* = 8 images, from 6-9 organoids, 1 cell line, 2 differentiation batches). Graph shows the percentage of PCNA-positive cells on total cells per organoid **(D)**. Representative fluorescence images of cortical organoid slices immunolabeled for proliferation marker PCNA (in red) and counterstained for cell nuclei with DAPI (in blue) **(E)**. Scale bar: 15 μm. **(F)** Quantification of cell density of cortical organoids expressed as number of cell nuclei per μm^2^ (Control and CDD conditions: *n* = 4-3 images, from 6-9 organoids, 1 cell line, 2 differentiation batches). Data are expressed as the average organoid cell density measured for each experimental replicate. *p < 0.05, **p < 0.01, ***p < 0.001 (Dunn’s test after Kruskal–Wallis test for data sets in **(B)**; Fisher’s LSD test after one-way ANOVA for data set in **(D)** and **(F)**).

As an advanced three-dimensional *in vitro* model, brain organoids replicate key aspects of brain development, providing valuable insights into cellular processes such as proliferation, survival, and neuronal differentiation in healthy and pathological contexts. Given the critical role of CDKL5 in these processes (Van Bergen et al., 2022; Katayama et al., 2020; Demarest ST. et al., 2019; Kind and Bird, 2021; Fuchs et al., 2014; Fuchs et al., 2019; Loi et al., 2020), we first assessed whether cell proliferation is affected in CDD organoids by examining the number of cells positive for the proliferation markers Ki-67 and PCNA (Scholzen and Gerdes, 2000; Celis and Celis, 1985). We found a lower Ki-67 signal intensity (Figures 4A–C) and fewer PCNA-positive cells (Figures 4D,E) in CDD organoids compared to controls, suggesting a reduced proliferation rate in the absence of CDKL5. Interestingly, while infection with the vector delivering native CDKL5 resulted in only a marginal recovery of cell proliferation (Figures 4B,D), both Ki-67 signal intensity and PCNA-positive cell number were restored in CDD organoids infected with the TATk-CDKL5 vector (Figures 4B,D) indicating a more robust recovery of the proliferative phenotype in organoids treated with the TATk-CDKL5 vector compared to those treated with the native CDKL5 vector. Because of reduced cell proliferation, CDD organoids also showed reduced cell density (Figure 4F) that was recovered only by expression of the TATk-CDKL5 protein (Figure 4F).

### 3.4 Effect of gene therapy with Igk-TATk-CDKL5 or CDKL5 vector on neuronal survival of CDD patient-derived organoids

**FIGURE 5:**
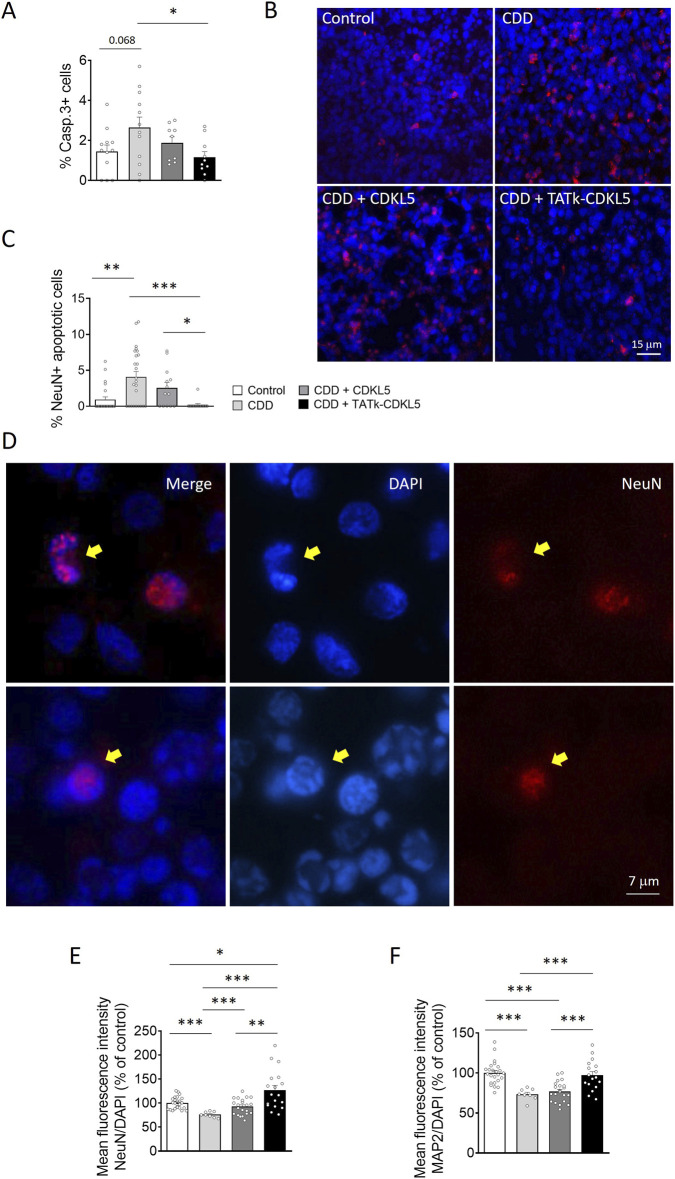
Neuronal survival in CDD cortical organoids following gene therapy. Evaluation of neuronal survival in CDD and control cortical organoids grown under constant agitation, treated at 11 weeks old with TATk-CDKL5 or CDKL5 gene therapy vectors at a dose of 3.6*10^10^ vg/org, or vehicle as a control, and collected 3 weeks after treatment for immunostaining. **(A)** Quantification of Cleaved Caspase-3-positive cells in cortical organoids treated with gene therapy vectors or vehicle as a control (Control and CDD conditions: *n* = 8–12 images, from 6-9 organoids, 1 cell line, 2 differentiation batches). Graph shows the percentage of Caspase-3-positive cells on total cells per organoid. **(B)** Representative fluorescence images of cortical organoid slices immunolabeled for Cleaved Caspase-3 (in red) and counterstained for cell nuclei with DAPI (in blue). Scale bar: 10 μm. **(C)** Quantification of apoptotic neurons in cortical organoids treated with gene therapy vectors or vehicle as a control (Control and CDD conditions: *n* = 13–25 images, from 6-9 organoids, 1 cell line, 2 differentiation batches). Graph shows the percentage of total NeuN-positive cells showing apoptotic nuclei. **(D)** Representative fluorescence images of cortical organoid slices immunolabeled for NeuN (in red) and counterstained for cell nuclei with DAPI (in blue). The yellow arrows indicate apoptotic NeuN-positive cells. Scale bar = 7 μm. **(E,F)** Quantitative analysis of NeuN **(E)** and MAP2 **(F)** intensity, normalized to DAPI intensity in cortical organoids treated with gene therapy vectors or vehicle as a control (Control and CDD conditions: *n* = 8–32 images, from 6-9 organoids, 1 cell line, 2 differentiation batches). Data sets in **(A,C,E,F)** are expressed as percentage, with data sets in **(E,F)** expressed in comparison to vehicle-treated control organoids. *p < 0.05, **p < 0.01, ***p < 0.001 (Fisher’s LSD test after one-way ANOVA, for data set in **(C)** and **(F)**; Unpaired t-test after Welch’s ANOVA, for data sets in **(A)** and **(E)**).

Previous studies have shown that CDKL5 deficiency reduces neuronal survival in vitro and *in vivo* models of CDD (Fuchs et al., 2014; Fuchs et al., 2019; Loi et al., 2020). Similarly, we found that cortical organoids from CDD patients are characterized by an increased number of cleaved caspase 3-positive cells, and pyknotic nuclei compared to control organoids (Figures 5A,B; Supplementary Figure S3A). Interestingly, while CDD organoids treated with a vector delivering native CDKL5 showed only a slight reduction in cell death, organoids infected with the TATk-CDKL5 vector showed a significant reduction in the number of caspase 3-positive cells and pyknotic nuclei, bringing them to levels similar to those observed in controls (Figures 5A,B; Supplementary Figure S3A).

Since active caspase-3 has been identified as a key mediator of apoptosis in neuronal cells (D'Amelio et al., 2010), confirming that CDKL5 deficiency predisposes neurons to cell death (Fuchs et al., 2019), we observed a higher number of neuronal cells (NeuN-positive) with nuclei exhibiting consistent structural changes characteristic of apoptotic nuclear condensation (Toné et al., 2007) in CDD organoids compared with control organoids (Figures 5C,D). Notably, only CDD organoids infected with the TATk-CDKL5 vector showed a significant recovery of neuronal survival (Figures 5C,D). Conversely, we did not observe significant differences in the number of proliferating cells undergoing apoptosis (PCNA+ and Casp3+ cells; Supplementary Figure S3B,C) between CDD and control organoids, indicating that CDKL5 deficiency selectively predisposes mature neurons, rather than proliferating cells, to apoptosis. Furthermore, we did not observe a rescue effect following gene therapy in this specific population (Supplementary Figure S3B).

In accordance with the reduced neuronal survival, CDD organoids displayed a lower immunostaining signal intensity of the neuronal markers NeuN and MAP2 compared with control organoids (Figures 5E,F; Supplementary Figure S4), suggesting a reduced number of neuronal cells in the absence of CDKL5. Consistently, only treatment with the TATk-CDKL5 vector, which rescued neuronal survival (Figures 5C,D), also restored NeuN and MAP2 signal intensity in CDD organoids (Figures 5E,F; Supplementary Figure S4). The slightly higher NeuN fluorescence intensity observed in TATk-CDKL5–treated organoids (Figure 5E) reflects natural variability in neuronal density across organoids and tissue sections and is attributed to rescued neuronal survival in CDD organoids rather than supra-physiological neurogenesis.

## 4 Discussion

Addressing key limitations of conventional gene therapies is essential to achieve efficient and widespread therapeutic delivery, particularly in disorders with complex and diffuse brain pathology, such as CDKL5 Deficiency Disorder (CDD). We developed a novel strategy based on a cross-correction mechanism that uses a secretable and cell-penetrating TATk-CDKL5 fused protein to enhance its distribution and functionality across the brain (Medici et al., 2022). While this innovative therapy showed promising results in a preclinical murine model (Medici et al., 2022), here we validated its therapeutic potential in a human-relevant model, CDD patient-derived cortical organoids, leveraging their ability to replicate the human disease phenotypes. Our findings provide proof-of-principle evidence that gene therapy employing the recombinant TATk-CDKL5 protein can offer improved functional outcomes compared to CDKL5 protein alone in CDKL5-deficient cortical organoids, consistent with the proposed cross-correction properties of the Igk-TATk-CDKL5 transgene. While the current data support the conclusion that TATk-CDKL5 offers superior phenotypic rescue under equivalent experimental conditions, we also acknowledge that more extensive biochemical mapping and dose–response analyses will be required to further dissect the underlying mechanisms and will be addressed in future studies.

As previously demonstrated (Cho et al., 2022), we showed that the neurotropic AAVPHP.B, which has widespread use for gene delivery in mouse models (Medici et al., 2022; Ittner et al., 2019), is also suitable for gene delivery in human cortical organoids. We selected this vector to maintain consistency with our previous *in vivo* studies (Medici et al., 2022; Medici et al., 2025) and to allow a direct comparison of CDKL5 *versus* TATk-CDKL5 efficacy across species. Although AAVPHP.B is not the optimal serotype for human CNS applications, it provides reliable and predictable transduction in cortical organoids (Cho et al., 2022), making it appropriate for mechanistic evaluation in this proof-of-principle study. Exploring more clinically relevant AAV variants with improved human tropism will be an essential step in future translational work.

In this study, PBS served as the disease baseline control. Because both therapeutic vectors share the same viral backbone and capsid, any nonspecific effects of AAV exposure would be expected to affect both treatment groups equally. This experimental design allowed us to isolate CDKL5-specific rescue effects, independent of vector-related contributions (see Figures 1D,E). Although the inclusion of an inert AAV control (e.g., AAVPHP.B-GFP) would represent an ideal additional comparator, future translational studies will incorporate such controls to further refine vector-related effects.

Remarkably, the absence of proper vascularization in cortical organoids severely limits viral penetration into deeper regions, largely confining transgene expression to the peripheral layers. As a consequence, efficient recovery of CDKL5 expression across the entire organoid is hindered, as indicated by the sparse CDKL5-positive cells detected after AAVPHP.B infection at the dose of 3.6 * 10^10^ vg/org. As a result, assays that primarily probe superficial regions, such as MEA recordings and immunohistochemical analyses, detect measurable improvements correlating with local CDKL5 expression, whereas bulk molecular readouts, including Western blot, which average signals from both transduced peripheral areas and poorly transduced inner regions, show a less robust recovery.

These differences highlight how distinct experimental readouts provide complementary information. Western blot analysis reflects total CDKL5 protein levels at the whole-organoid level, whereas HA immunofluorescence reports expression at the cellular level within HA-positive cells. Moreover, for the secretable TATk-CDKL5 protein, partial extracellular release and redistribution through cross-correction are expected to reduce intracellular protein accumulation detectable by Western blot, despite comparable cellular expression revealed by HA fluorescence intensity. Direct visualization of soluble protein secretion and cross-correction in this system remains constrained by the genetic nature of the CDD model, as the R59X mutation abolishes CDKL5 protein expression without eliminating the endogenous CDKL5 transcript, preventing the use of CDKL5 mRNA-based approaches to distinguish non-transduced from cross-corrected cells.

Despite these limitations, CDKL5 re-expression resulted in a detectable recovery of EB2 phosphorylation, indicating partial reactivation of downstream signaling pathways, although complete normalization was not achieved. At present, spatial resolution of EB2 pathway activation within cortical organoids is limited, as available antibodies against total and phosphorylated EB2 do not provide reliable immunohistochemical staining in fixed tissue sections. Consequently, EB2 phosphorylation could be robustly assessed only by Western blot, which captures average pathway activation across the entire organoid but cannot resolve layer-specific effects. Improving transgene distribution throughout the organoid, potentially through vascularization strategies, will be essential to fully assess molecular rescue at the whole-organoid level. Future studies incorporating region- and cell-type-specific analyses, including quantitative assessment of vector genome copy number and CDKL5 mRNA expression, will be critical to refine dose–response relationships and further advance the translational potential of this approach.

Notably, assessment of electrical activity of the organoids through planar MEAs, which predominantly sample superficial regions, as discussed above, showed evidence of functional improvement. At the time of infection (11 weeks) and analysis (13–14 weeks), cortical organoids are expected to contain maturing neurons and radial glia–like neural progenitors, together with emerging early glial populations, consistent with established developmental timelines for human cortical organoids (Negraes et al., 2021; Trujillo et al., 2019). As reported in previous studies (Negraes et al., 2021; Wu et al., 2022), we found electrophysiological properties to be significantly impaired in CDD organoids compared to controls, reflecting pathological network hyperexcitability. Consistently, MEA recordings revealed increased electrical activity in CDD cortical organoids compared to controls. Interestingly, we observed a slight, but promising amelioration of the phenotype in TATk-CDKL5 gene therapy-treated CDD organoids that was not detectable in those treated with the CDKL5 alone, especially regarding network hyper-synchronization and firing activity. While signs of hyperexcitability still persist after treatment, the observed amelioration in electrical activity provides a promising initial indication of functional improvement, suggesting that the TATk-CDKL5 gene therapy may exert a stronger effect also on neuronal network activity compared to conventional CDKL5 delivery. Previous work has shown that CDD organoids display altered network activity within a narrow temporal window (12–16 weeks), with differences in spike frequency and synchronization diminishing at later stages, likely due to compensatory remodelling (Negraes et al., 2021; Wu et al., 2022). For this reason, we compared treated and untreated organoids within this window to obtain reliable measurements of immediate gene therapy effects. Given the short evaluation window of only 2 weeks post-infection, we cannot exclude that a more pronounced rescue may emerge with longer culture periods, allowing the organoids to further mature structurally and develop their networks in the presence of CDKL5. Future investigations using additional functional assays and longitudinal studies will be essential to achieve a more comprehensive phenotypic assessment of CDD cortical organoids and, consequently, a more accurate evaluation of therapeutic efficacy.

Extensive work in CDD has provided fundamental characterization of CDKL5 functionality, revealing its critical role in neuronal proliferation, differentiation, and survival *in vitro* and *in vivo* CDD models (Van Bergen et al., 2022; Katayama et al., 2020; Demarest ST. et al., 2019; Kind and Bird, 2021; Fuchs et al., 2014; Fuchs et al., 2019; Loi et al., 2020). It has been reported that 2D cultures of neural progenitors generated from CDD-iPSCs have proliferation defects and increased cell death (Negraes et al., 2021), while differentiated CDD neurons show cellular and morphological defects (Negraes et al., 2021). However, the impact of CDKL5 deficiency on cells organized in 3D cortical organoids has remained poorly understood. Here, we confirm the importance of CDKL5 in the correct developmental progression of cortical organoids. Our analysis revealed a significant reduction in cell proliferation within CDD organoids, as indicated by decreased Ki-67 signal intensity and fewer PCNA-positive cells than controls. The reduced proliferation corresponded with decreased overall cell density in CDD organoids. These findings suggest that CDKL5 deficiency impairs the proliferative capacity of progenitor cells, potentially contributing to abnormal organoid development. Since PCNA acts as a cofactor of DNA pol δ for DNA synthesis during not only DNA replication but also DNA repair (Maga and Hubscher, 2003), PCNA expression in cortical organoids may also indicate the activation of pro-survival repair mechanisms. Given the well-established link between apoptosis and unresolved DNA damage, the failure of PCNA recovery in CDD cortical organoids may reflect the inability of CDKL5 alone to rescue apoptosis, potentially explaining the different CDKL5 and TATk-CDKL5 treatment impact on the two proliferation markers, Ki-67 and PCNA. Further analyses using cell-type-specific proliferation markers could provide a more detailed understanding of which cell type, stem cells or neural progenitors, is more affected by CDKL5 loss. Moreover, while this restricted set of acute markers (caspase activation, nuclear morphology, proliferation) provided an informative overview also on treatment safety, a more comprehensive toxicity assessment will be needed in future work before translating to patients.

CDKL5 deficiency is also known to impair neuronal survival, as evidenced by increased apoptosis in both *in vitro* and *in vivo* models of CDD (Fuchs et al., 2014; Fuchs et al., 2019; Loi et al., 2020). For instance, *Cdkl5*-KO mice are characterized by an increased rate of apoptotic cell death in the hippocampal dentate gyrus that causes a reduction in the final number of granule neurons (Fuchs et al., 2014) and accelerated neuronal senescence/death during aging (Gennaccaro et al., 2021). Consistent with these findings, CDD cortical organoids exhibited a significant increase in cleaved caspase-3-positive cells, a hallmark of apoptosis, compared to control organoids. In addition, structural features indicative of apoptotic nuclear condensation were more frequently observed in NeuN-positive neurons from CDD organoids. This enhanced apoptotic activity highlights the vulnerability of neuronal populations in the absence of functional CDKL5, further confirming the pro-survival role of CDKL5. Recent studies have demonstrated that neurons lacking CDKL5 accumulate increased DNA damage and display enhanced sensitivity to neurotoxic stress (Fuchs et al., 2019; Loi et al., 2020), implicating CDKL5 in the DNA damage response through the interaction with chromatin remodelling and transcriptional regulation factors (Fuchs et al., 2019; Loi et al., 2020; Khanam et al., 2021). Indeed, although CDD is considered a neurodevelopmental disorder and not a neurodegenerative pathology based on preclinical studies performed on mouse models of the disease (Amendola et al., 2014; Wang et al., 2012; Okuda et al., 2018; Tang et al., 2019; Yennawar et al., 2019), recent studies indicate a progression of brain atrophy in CDD patients that could be attributable to a neurodegenerative phenomenon (Specchio et al., 2023). Future studies aimed at assessing alterations in DNA repair processes in CDKL5-deficient cortical organoids could help confirm the involvement of CDKL5 in genome maintenance and clarify the consequences for post-mitotic neurons, thereby improving our current understanding of the molecular and cellular mechanisms underlying CDD pathogenesis.

Consistent with a diminished neuronal population due to increased apoptosis and impaired proliferation, we found that CDD organoids showed lower immunostaining intensity for the neuronal markers NeuN and MAP2. This reduction may also reflect a reduced neuronal complexity and maturation, due to the critical role of CDKL5 in neuronal development and differentiation, which has already been extensively highlighted in both *in vitro* and *in vivo* models of CDD (Van Bergen et al., 2022; Amendola et al., 2014; Wang et al., 2012; Fuchs et al., 2014; Valli et al., 2012; Chen et al., 2010; Ricciardi et al., 2012; Zhu et al., 2013; Pizzo et al., 2016; Kontaxi et al., 2023; Nawaz et al., 2016). In accordance with other results, the enhanced recovery of these structural deficits observed in CDD organoids expressing the recombinant TATk-CDKL5 protein further supports its increased therapeutic efficacy compared to CDKL5 alone.

## 5 Conclusion

In this work, we have confirmed the pro-proliferative and pro-survival role of CDKL5 in a human-derived model that recapitulates early brain development. These findings highlight the value of cortical organoids as a model to explore complex cellular and molecular mechanisms underlying CDD. The use of human cells provides strong translational relevance, enabling the evaluation of therapeutic responses within a genetic and physiological context closely aligned with patients. However, we are aware that iPSC-derived organoids do not fully replicate the complexity of *in vivo* neural circuits, nor the influence of systemic factors present in the brain, underscoring the need for further evaluations to support future clinical translatability. Nevertheless, compared with animal models, human iPSC-derived organoids offer a more accurate and human-specific framework for assessing therapeutic efficacy and safety, thus representing a valuable tool for preclinical validation.

Using cortical organoids derived from CDD patients, we obtained additional evidence supporting the superior efficacy of the recombinant TATk-CDKL5 protein-based gene therapy compared with conventional CDKL5 delivery. This work provides the first proof of principle for the therapeutic potential of TATk-CDKL5 gene therapy in patient-derived human neural systems. Results obtained so far in a mouse model of CDD (Medici et al., 2022), and now in CDD patient-derived cortical organoids, indicate that the TATk-CDKL5 gene therapy is more effective in improving the pathological phenotype in CDKL5-deficient cells based on the unique properties of the Igk-TATk fusion peptide.

Future efforts to improve viral delivery, enhance transgene expression across neuronal populations, and assess additional structural outcomes, such as neuronal connectivity, will be essential to refine and expand evidence of the therapeutic potential of this approach. While our present and past studies have focused on short-term outcomes, long-term investigations will also be critical to establish whether the observed improvements translate into durable effects on neuronal maturation, synaptic organization, and functional integration.

## Data Availability

The raw data supporting the conclusions of this article will be made available by the authors, without undue reservation.
