# Supplementary material for: A cross-correction gene therapy approach for CDKL5 deficiency disorder improves the pathological phenotype of CDD patient-derived cortical organoids
Source: Front Bioeng Biotechnol. 2026 Jan 21;13:1744903. doi: 10.3389/fbioe.2025.1744903 (PMC12868234; doi:10.3389/fbioe.2025.1744903)
Supplement: Supplementary file 1 [file Supplementaryfile1.pdf]

Supplementary Material

1 Supplementary Figures and Tables

1.1 Supplementary Figures

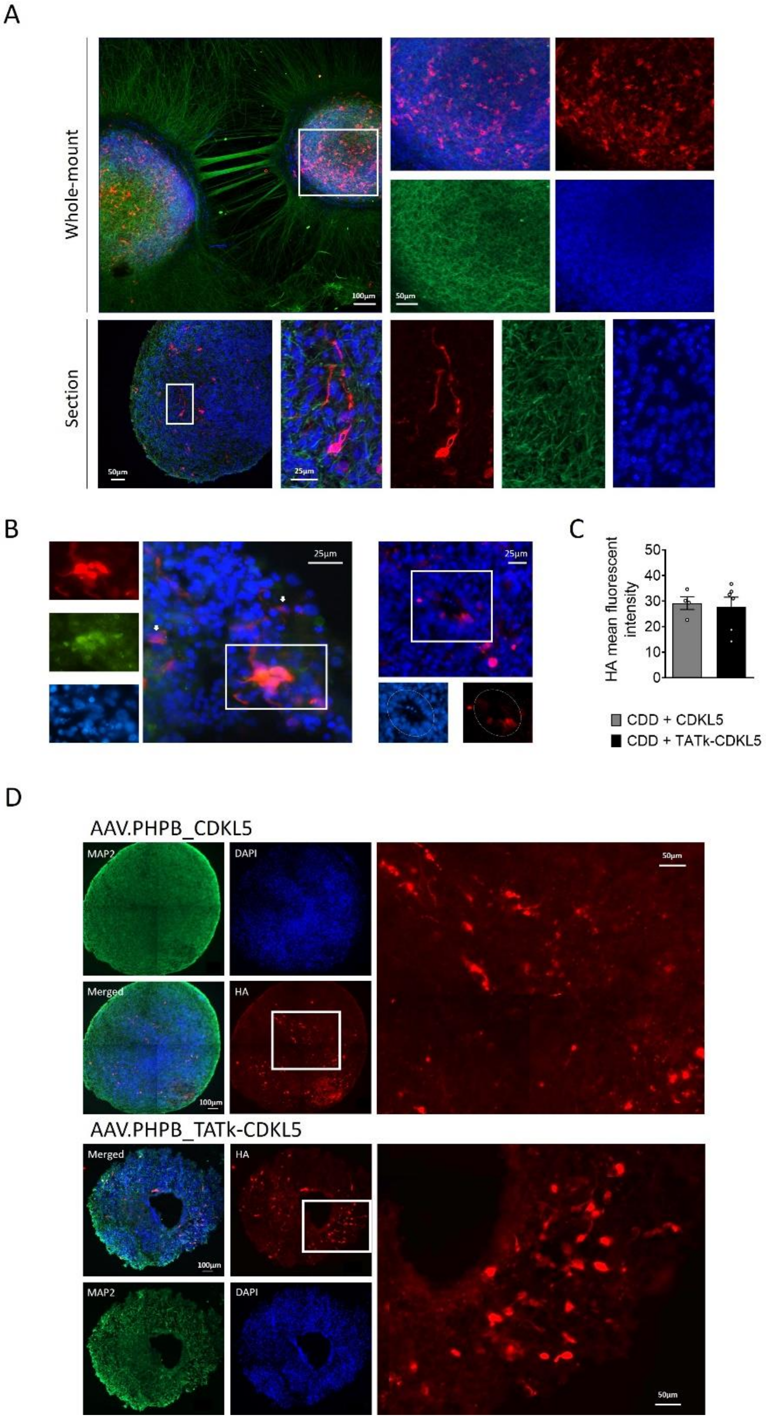

Supplementary Figure 1. CDKL5 protein expression in CDD cortical organoids following gene therapy. (**A**) Representative fluorescence images of whole-mount immunostaining (upper panels) and a cortical organoid slice (lower panels) showing CDKL5 expression (HA, in red) in 11-week-old control cortical organoids infected with TATk-CDKL5 gene therapy vector at the dose of $7.2*10^{10}$ vg/org and collected 1 week after infection; MAP2 (in green) and nuclei counterstained with DAPI (in blue). White rectangles indicate the region shown in the high-magnification images on the right. Scale bar = 100 µm and 50 µm (low magnification); 50 µm and 25 µm (high magnification). (**B**) Representative fluorescence images of cortical organoid slice showing different cell subtypes expressing CDKL5 protein (HA, in red) in control cortical organoids infected at 11 weeks old with TATk-CDKL5 gene therapy vector at the dose of $3.6*10^{10}$ vg/org and collected 3 weeks after infection for immunostaining. Left panels show CDKL5 expression in differentiated neurons (NeuN-positive cells, in green); white arrows indicate HA-positive non-neuronal cells (NeuN-negative). White rectangles indicate the region shown in the magnification images. Right panels show a rosette with progenitor cells expressing CDKL5 protein. The dotted outline delineates rosette structure. Nuclei counterstained with DAPI (in blue). Scale bar = 25 µm. (**C**) Quantification of HA mean intensity in organoid slices; organoids were infected as described in (D). Values are represented as means ± SEM. (Unpaired t-test). (**D**) Representative fluorescence images of CDD cortical organoid slices showing distribution of TATk-CDKL5 or CDKL5 expression (HA, in red) in CDD cortical organoids infected at 11 weeks old with TATk-CDKL5 or CDKL5 gene therapy vector at a dose of $3.6*10^{10}$ vg/org and collected 3 weeks after infection for immunostaining; MAP2 (in green) and nuclei counterstained with DAPI (in blue). The boxed areas indicate representative HA-positive cells; higher-magnification images show a punctate intracellular HA signal corresponding to CDKL5 protein within infected cells. Scale bar = 100 µm (low magnification); 50 µm (high magnification).

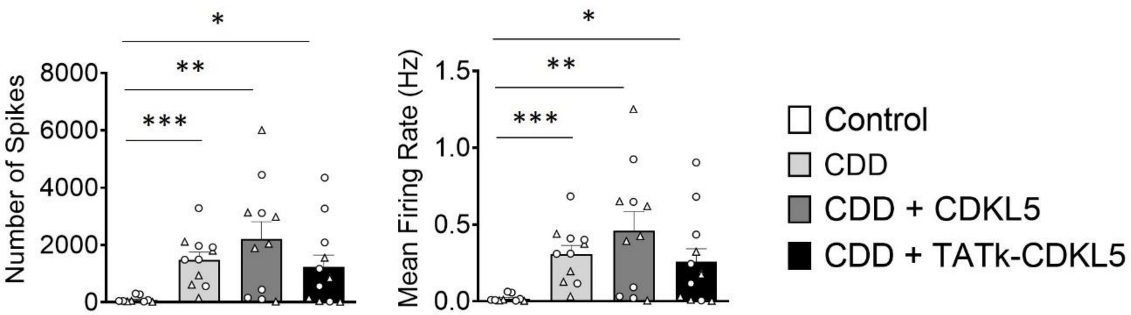

Supplementary Figure 2. **Electrophysiological recordings in CDD cortical organoids following gene therapy.** Electrophysiological recording of CDD and control cortical organoids grown on MEA plates, treated at 11 weeks old with TATk-CDKL5 or CDKL5 gene therapy vectors at a dose of $3.6*10^{10}$ vg/org, or vehicle as a control. Electrical activity was recorded 2 weeks after treatment. Graphs show the result of several electrical parameters as indicated during 3 minutes of recording (Control and CDD conditions: *n* = 9-12 wells, 3 org per well; 2 cell line, 1 differentiation batch). Dots and triangles in the scatter plots identify the two independent CDD iPSC lines. Values are represented as means ± SEM. * $p < 0.05$, ** $p < 0.01$, *** $p < 0.001$ (Unpaired t-test after Welch's ANOVA).

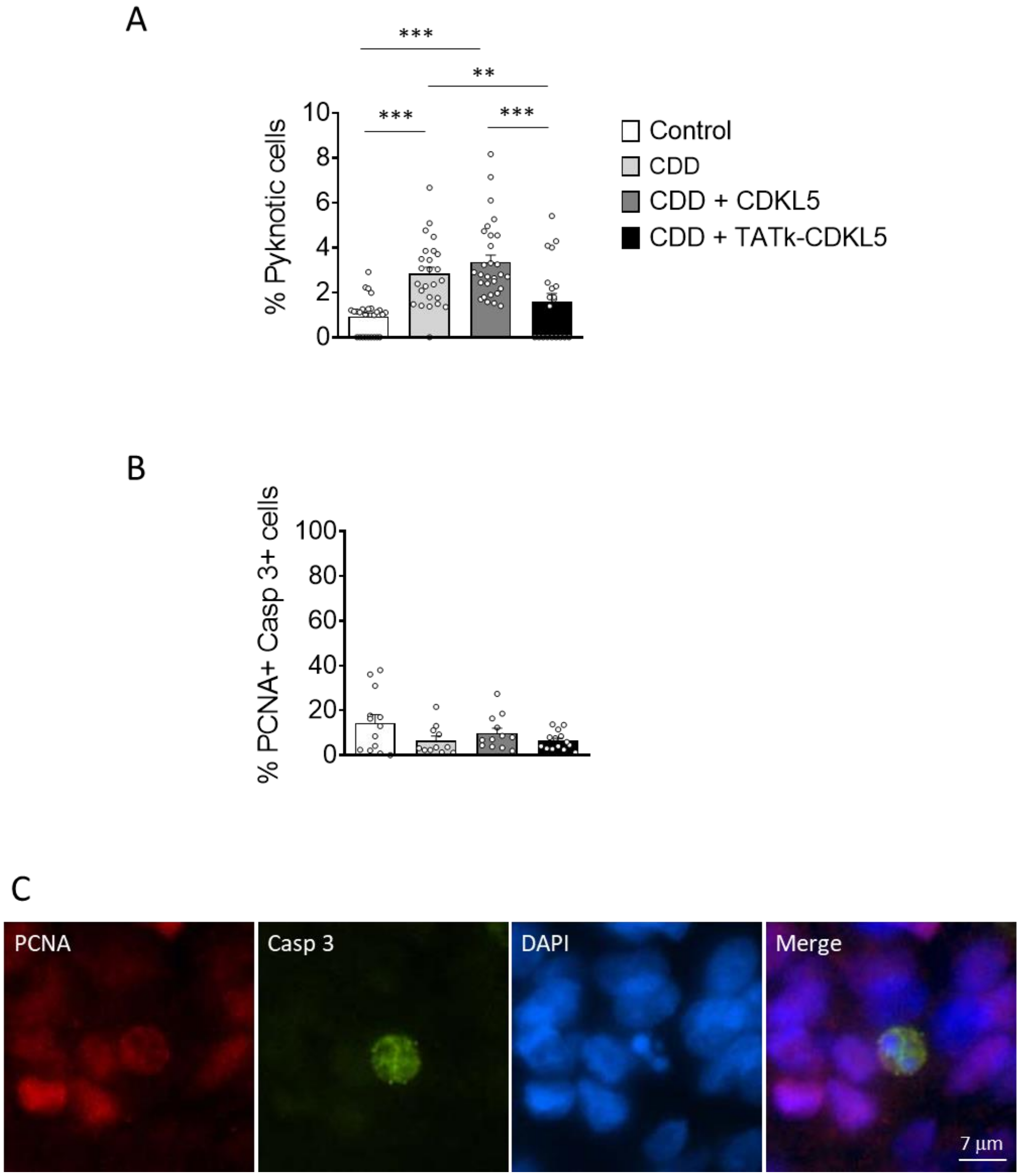

Supplementary Figure 3. Cell death in CDD cortical organoids following gene therapy. (A) Graph shows pyknotic nuclei quantification in cortical organoids following gene therapy (Control and CDD conditions: n = 15-30 images, from 6 organoids, 1 cell line). (B) Graph shows the percentage of PCNA-positive cells that are also positive for cleaved caspase-3 (Control and CDD conditions: n = 12-15 images, from 6 organoids; 1 cell line, 2 differentiation batches). (C) Representative fluorescence images of a cortical organoid slice immunolabeled for PCNA (in red) and cleaved Caspase-3 (in green), with cell nuclei counterstained using DAPI (in blue). Scale bar = 7 µm. Data in A and B are expressed as mean ± SEM. ** $p < 0.01$, *** $p < 0.001$ (Dunn's test after Kruskal-Wallis).

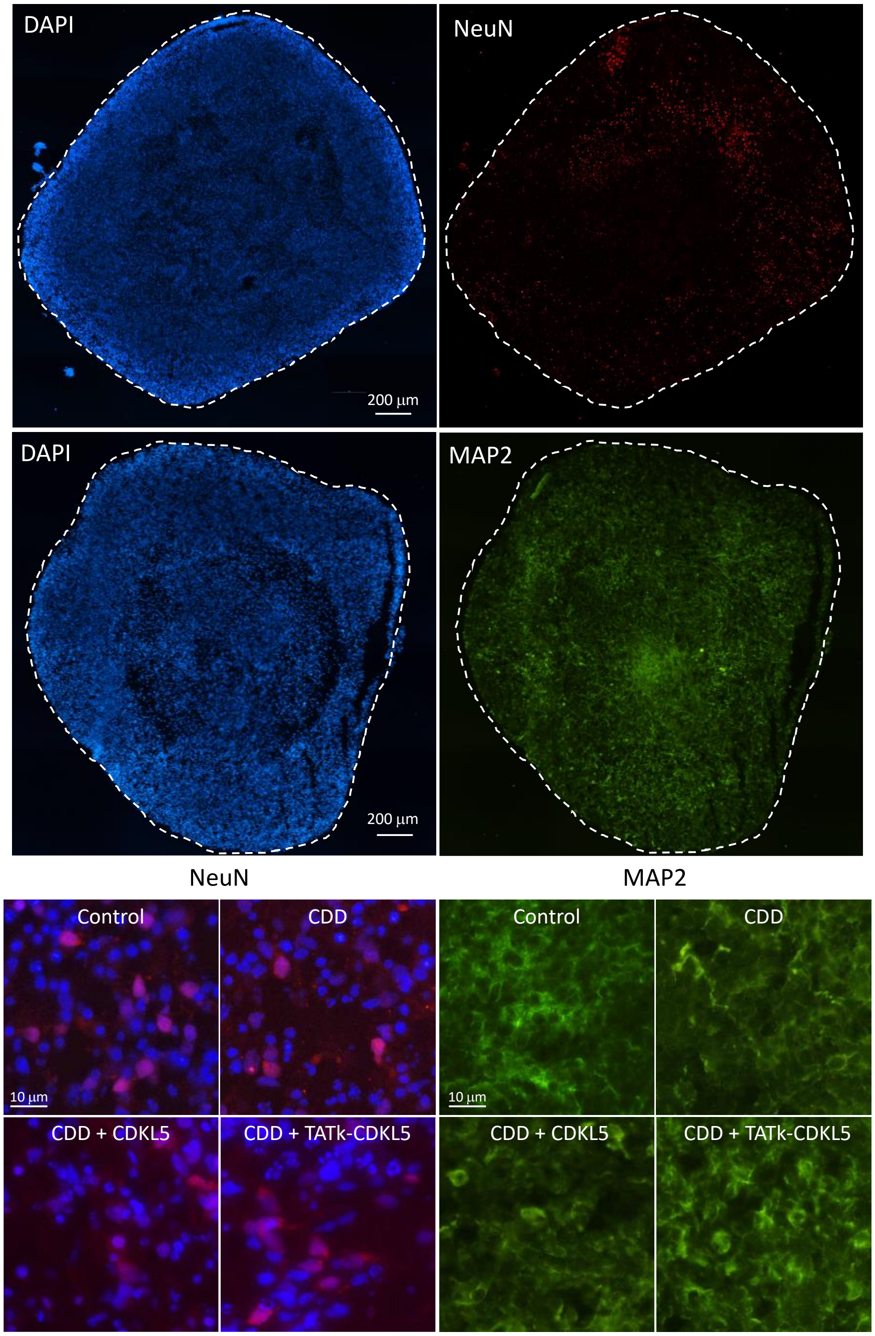

Supplementary Figure 4. Neuronal complexity and maturation of CDD cortical organoids following gene therapy. Representative fluorescence images of CDD and control cortical organoid slices showing the expression of the neuronal markers NeuN or MAP2, as indicated, in cortical organoids treated at 11 weeks old with TATk-CDKL5 or CDKL5 gene therapy vectors at a dose of $3.6*10^{10}$ vg/org, or vehicle as a control, and collected 3 weeks after treatment for immunostaining; nuclei were counterstained with DAPI (in blue). Upper panels show lower magnification images with dotted lines indicating the manually traced area of organoids in which signal intensity was quantified, while lower panels show representative high-magnification images. Scale bar = 200 µm (low-magnification); 10 µm (high-magnification).

1.2 Supplementary Tables

Supplementary Table 1. Primary and secondary antibodies used for Western blot and Immunostaining.

Antibody	Company	Dilution	Code
Immunohistochemistry			
**HA**	Cell Signaling Technologies	1:500	#3724
**Ki-67**	Invitrogen	1:200	#MA5-14520
**PCNA**	Sigma	1:500	#P8825
**NeuN**	Millipore	1:250	#MAB377
**MAP2**	Millipore	1:500	#AB5622
**Cleaved Caspase 3**	Cell Signaling Technologies	1:200	#9661S
**Goat anti-Rabbit IgG- Alexa Fluor 555**	Invitrogen	1:500	#A-21428
**Goat anti-Mouse IgG- Alexa Fluor 555**	Invitrogen	1:500	#A-21422
**Donkey anti-Rabbit IgG- Alexa Fluor 488**	Invitrogen	1:500	#A-21206
Western blot			
**hCDKL5**	Santa Cruz	1:500	#sc-376314
**EB2**	Abcam	1:1000	#ab234843
**P(Ser222)-EB2**	Covalab	1:1000	#pab01032-P
**B-Actin**	Abcam	1:1000	#8226
**IRDye 680RD Goat anti-Mouse IgG**	LICORbio	1:5000	#926-68070
**IRDye® 800CW Goat Anti-Rabbit IgG**	LICORbio	1:5000	#926-32211

Supplementary Table 2. Descriptive statistics.

Figure	*Test*	*Format*	*Values*	*Significance*
Fig. 1C	Two-Way ANOVA	F (DFn, DFd)	35,74 (1, 39)	$p < 0.0001$
Fig. 1D	Unpaired t test	t (df)	0.2703 (8)	$p = 0.7938$
Fig. 2C	Welch's ANOVA	W (DFn, DFd)	33.55 (2, 5.088)	$p = 0.0012$
Fig. 2E	Welch's ANOVA	W (DFn, DFd)	9.584 (3, 5.712)	$p = 0.0118$
Fig. 3B (Burst)	Welch's ANOVA	W (DFn, DFd)	5.804 (3, 21.57)	$p = 0.0046$
Fig. 3B (Network burst)	Kruskal-Wallis test	H (DF)	6.491 (3)	$p = 0.0900$
Fig. 3B (Synchrony)	Welch's ANOVA	W (DFn, DFd)	2.561 (3, 22.68)	$p = 0.0801$
Fig. 4B	Kruskal-Wallis test	H (DF)	24.16 (3)	$p < 0.0001$
Fig. 4D	One-Way ANOVA	F (DFn, DFd)	5.040 (3, 28)	$p = 0.0064$
Fig. 4F	One-Way ANOVA	F (DFn, DFd)	3.516 (3, 10)	$p = 0.0569$
Fig. 5A	Welch's ANOVA	W (DFn, DFd)	2.347 (3, 21.44)	$p = 0.1012$
Fig. 5C	Kruskal-Wallis test	H (DF)	18.51 (3)	$p = 0.0003$
Fig. 5E	Welch's ANOVA	W (DFn, DFd)	23.03 (3, 33.68)	$p < 0.0001$
Fig. 5F	One-Way ANOVA	F (DFn, DFd)	14.23 (3, 66)	$p < 0.0001$
Sup. Fig. S1D	Unpaired t test	t (df)	0.7684 (29)	$p = 0.4485$
Sup. Fig. S2 (Spikes)	Welch's ANOVA	W (DFn, DFd)	14.48 (3, 17.54)	$p < 0.0001$
Sup. Fig. S2 (Firing rate)	Welch's ANOVA	W (DFn, DFd)	14.48 (3, 17.54)	$p < 0.0001$
Sup. Fig. S3A	Kruskal-Wallis test	H (DF)	40.45 (3)	$p < 0.0001$
Sup. Fig. S3B	Kruskal-Wallis test	H (DF)	3.239 (3)	$p = 0.3562$
